# Novel Method for High-Throughput Full-Length IGHV-D-J Sequencing of the Immune Repertoire from Bulk B-Cells with Single-Cell Resolution

**DOI:** 10.3389/fimmu.2017.01157

**Published:** 2017-09-14

**Authors:** Stefano Vergani, Ilya Korsunsky, Andrea Nicola Mazzarello, Gerardo Ferrer, Nicholas Chiorazzi, Davide Bagnara

**Affiliations:** ^1^Karches Centre for Chronic Lymphocytic Leukemia Research, The Feinstein Institute for Medical Research, Northwell Health, Manhasset, NY, United States; ^2^Hofstra-Northwell Health School of Medicine, Hempstead, NY, United States; ^3^Robert S. Boas Center for Genomics & Human Genetics, The Feinstein Institute for Medical Research, Northwell Health, Manhasset, NY, United States; ^4^Department of Experimental Medicine, University of Genoa, Genoa, Italy

**Keywords:** next generation sequencing, immunoglobulin repertoire, Illumina Miseq sequencing, VDJ rearrangement, cDNA library, unique molecular identifier, B lymphocytes

## Abstract

Efficient and accurate high-throughput DNA sequencing of the adaptive immune receptor repertoire (AIRR) is necessary to study immune diversity in healthy subjects and disease-related conditions. The high complexity and diversity of the AIRR coupled with the limited amount of starting material, which can compromise identification of the full biological diversity makes such sequencing particularly challenging. AIRR sequencing protocols often fail to fully capture the sampled AIRR diversity, especially for samples containing restricted numbers of B lymphocytes. Here, we describe a library preparation method for immunoglobulin sequencing that results in an exhaustive full-length repertoire where virtually every sampled B-cell is sequenced. This maximizes the likelihood of identifying and quantifying the entire IGHV-D-J repertoire of a sample, including the detection of rearrangements present in only one cell in the starting population. The methodology establishes the importance of circumventing genetic material dilution in the preamplification phases and incorporates the use of certain described concepts: (1) balancing the starting material amount and depth of sequencing, (2) avoiding IGHV gene-specific amplification, and (3) using Unique Molecular Identifier. Together, this methodology is highly efficient, in particular for detecting rare rearrangements in the sampled population and when only a limited amount of starting material is available.

## Introduction

The diversity of the adaptive immune system is the key to its ability to respond to a wide variety of antigens. Extensive knowledge of the adaptive immune receptor repertoire (AIRR) could have a major impact on basic and translational research since it can help to better understand the dynamics and diversity of the AIRR, study immune responses induced by vaccines and infectious agents, and determine minimal residual disease, intra-clonal diversity, and evolution in lymphoma/leukemia. Recent advances in next generation sequencing allow in-depth studies of AIRR of B (Ig-seq) and T lymphocytes.

B lymphocytes originate in the bone marrow where precursors pass through a series of highly regulated processes to generate a functional B-cell receptor that is necessary for the survival of mature B cells (1). In humans, the variable region of the IGH chain is created by the recombination of one of ~50 variable (IGHV) genes, one or more of ~30 diversity (IGHD), and one of 6 joining (IGHJ) genes. Within the recombined IGHV-D-J, the CDR3 is the most variable segment and is the major contributor for antigen contact and the antigen-binding site. Its variability is increased as a consequence of imperfect joining with random nucleotide insertion and deletions occurring during the recombination process. This yields an antigen-inexperienced B cell with a virtually unique IGHV-D-J rearrangement, without a germline reference and, therefore, a characteristic antigen-binding site. Finally, the diversity and complexity of the AIRR obtained by recombination is further increased in secondary lymphoid tissues by another biological process termed somatic hypermutation, whereby the enzyme activation-induced deaminase introduces mutations in the rearranged IGHV-D-J.

Determining the DNA sequence of the AIRR presents major challenges compared to targeted sequencing of other genes because of its linkage, at the mRNA level, to the constant region of IGH. The latter can change with B-lymphocyte maturation, moving from IgM to IgM + IgD to non-IgM (IgG, IgA, and IgE) isotypes. In addition, AIRR DNA sequencing is made even more challenging because of the absence of germline reference for the VH CDR3. This makes reliably reconstructing the sequences from short reads challenging, although certain library preparations have successfully addressed this problem (2). Finally, the extent of *in vivo* repertoire diversity and limited biological sampling (3) pose major problems, especially in human studies. In addition, the full diversity of the already limited sampled material is often not reflected leading to poor repertoire overlapping of technical replicates (4). It is, therefore, important and challenging to obtain a comprehensive repertoire representation of as many sampled cells as possible.

In studies where attention is focused on expanded B-lymphocyte clones, such as an immune response to a specific environmental insult, overrepresented rearrangements can be easily detected. On the other hand, in cases where attention is focused on non-expanded/rare cells (i.e., naïve, immature, and long-term memory B cells, or in detection of minimal residual disease in the context of a B-cell malignancy), the resulting repertoire is far more susceptible to biases intrinsic to methodology. Biases often result from amplicon length in the case of template-switch PCR, and in IGHV gene-specific primer for multiplex PCR. Despite bias correction to obtain quantitative data can be performed (5), it is not possible to recovery rearrangements that have not been detected in the sequencing process. Since high yields are crucial to obtain a reasonable representation, this poses challenges, especially when dealing with B-cell fractions from a limited sample. For example with the template-switch method, efficiency is less than 1 molecule per naïve B cell (6).

In light of these issues, we present a method that allows Ig-seq of the full IGHV-D-J-CH transcript, with single-cell resolution, from a pool of B cells.

## Results

### Library Preparation

**Figure 1:**
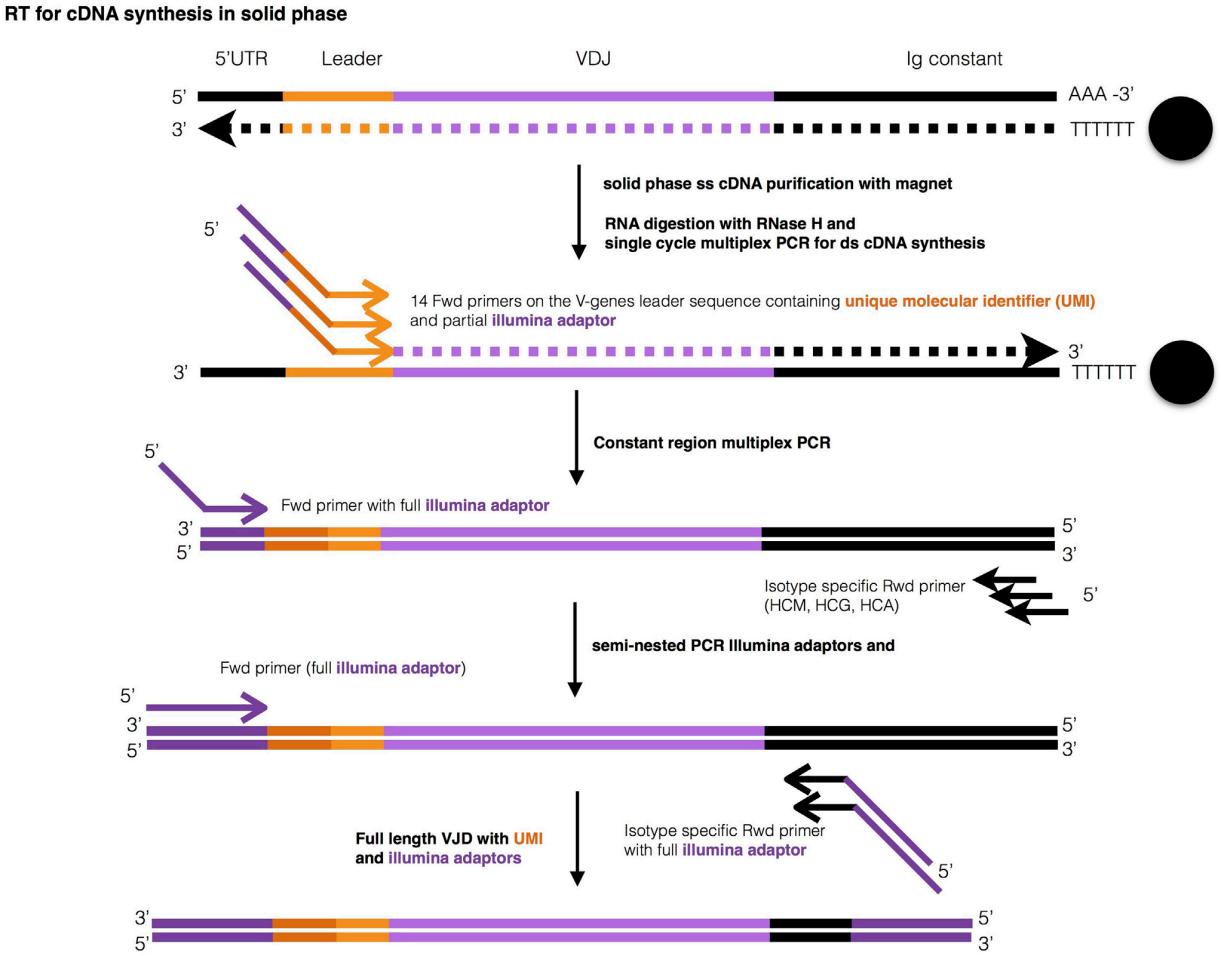
Graphic representation of library preparation. Ig-seq library preparation consists of 4 steps: (1) mRNA purification and reverse transcription, (2) IGH-specific double strand cDNA synthesis with multiplex primers, both while bound to poly-T magnetic beads, () two step semi-nested PCR, and (1) Illumina indexing.

A defined number of B lymphocytes (<100–25,000) were sorted directly into 200 µl PCR tubes containing cell lysis buffer, and mRNA was isolated using poly-T coupled to magnetic beads (Figure 1). The entire amount of isolated mRNA was reverse transcribed in this solid phase, with the poly-T stretch working as primer for the reaction. Beads containing the resultant single-stranded cDNA were then purified with a magnet, and cDNA was used for the synthesis of double-strand (ds) cDNA of the IGHV-D-J rearrangements employing multiplex primers annealing to the 5’ of the leader sequence (Table S1 in Supplementary Material). During ds-cDNA synthesis, a unique molecular identifier (UMI) consisting of 13–16 random nucleotides and containing in addition a partial Illumina adaptor, were introduced into each second strand of the cDNA. Then, the IGHV-D-J-CH ds-cDNA—purified by a magnet as above—was used to perform PCR amplification with a universal forward primer and a mix of CH isotype-specific reverse primers. The PCR product was used as a template for a semi-nested PCR with inner CH primers that allowed introduction of partial Illumina adaptors, which were used for library indexing.

In each of the above steps until the first PCR amplification, the entirety of mRNA, cDNA, and ds-cDNA was used, never being diluted. After each step, the material obtained was washed while attached to the original set of beads and resuspended directly in the reaction buffer of the following step. This poly-T magnetic bead purification of mRNA and of cDNA (purification for ds-cDNA not tested) was at least 7-times more efficient than a column-based method when tested on a starting material of 100,000 cells (data not shown). Moreover, the column-based method gave inconsistent results when starting from less than 10,000 cells, indicating even lower efficiency (not shown). Finally, our method was easily carried out in 96-well plates. The indexed library was sequenced with Illumina MiSeq v3 (600 cycles).

### Raw Sequence Analysis and Error Correction

The defined raw Illumina sequences were analyzed using pRESTO tools (7). Sequences were clustered based on UMI identity (allowing one error in the UMI region). Only those sequences having at least 90% identity in the first 150 nt (region with higher quality) and including the HCDR3 on both Illumina reads from each UMI group (UMIG) were used to build a single consensus sequence; this step compensated for possible errors in the UMI region and for independent molecules that could be tagged erroneously with the same UMI. For analysis, we used sequences for which the consensus was obtained by matching at least three reads or by three identical sequences from different UMIGs.

### Effects of Read Clustering

**Figure 2:**
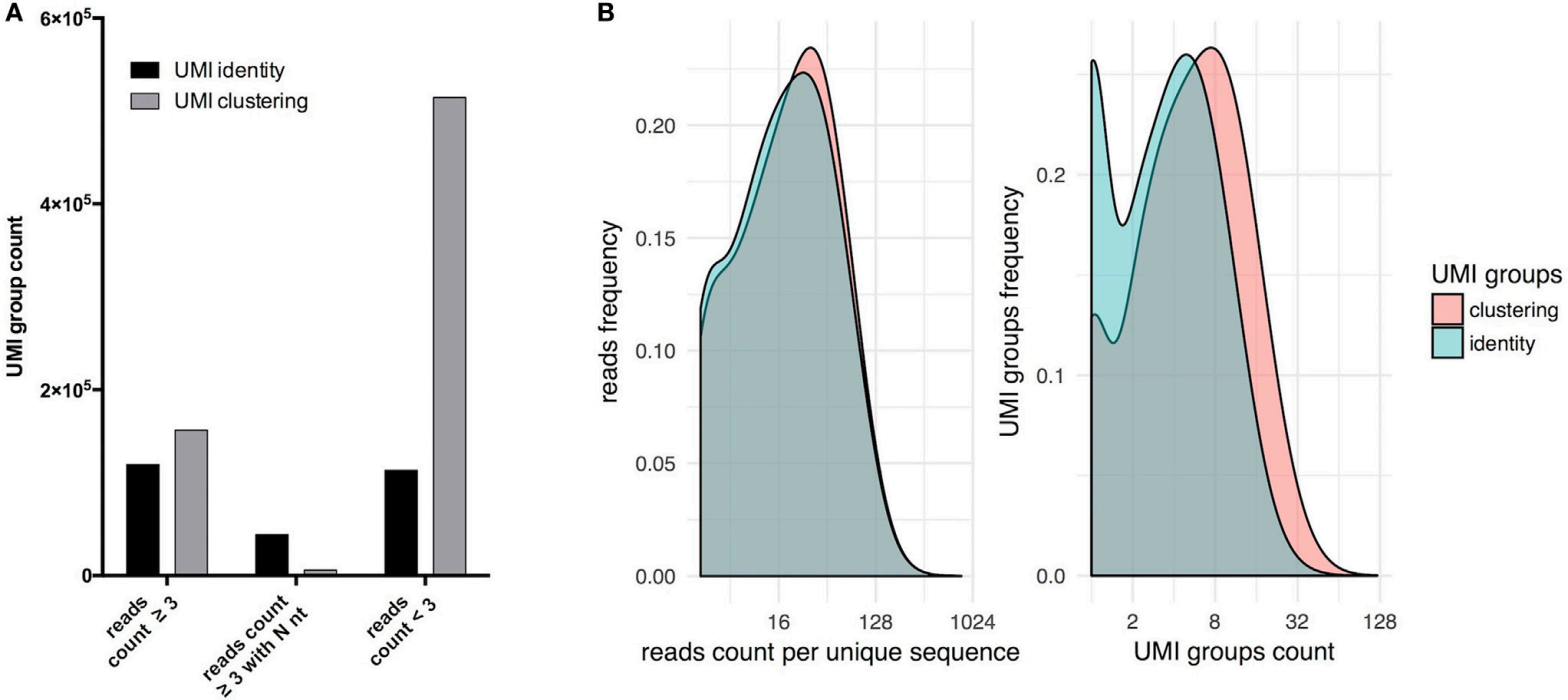
Forming unique molecular identifier (UMI) groups by sequence clustering increases the number of sequences passing filter. **(A)** Allowing one error in the UMI sequence increases the sequences deriving from three or more raw reads compared to the grouping of UMI by read identity. In addition, this leads to a decrease in the number of ambiguous calls (N nt) during consensus assignment. **(B)** Sequences deriving from UMI groups by sequence clustering have higher UMI counts.

At a sequencing depth of 40× per starting cell for PBMC-derived naïve B cells, analyses were performed as described above with UMI and read clustering with regular UMI grouping solely by identity (Figure 2). Upon clustering, the total number of UMIGs increased more than twofold, the number of UMIGs passing the filter increased by 30%, and the sequences belonging to a group composed of at least three independent reads increased by 300% (Figure 2A). After consensus filtering (Figure 2B), the mean read count per unique sequence increased from 29 to 31, and the mean UMIG count per unique sequence increased from 4.7 to 7.6; concomitantly, sequences with UMIG counts equal to one decreased from ~6 to ~1.8%. Clustering increased significantly singletons, which result from removing unrelated sequences with the same UMIG, and lead to less ambiguous nucleotide calls in forming the consensus sequence. After paired-end assembly, the presence of sequences containing an N nucleotide derived from the consensus of three or more reads was ~7 times lower when UMI and read clustering were performed (Figure 2A).

### Measurement of Specific IGHV Gene Detectability

To estimate the ability to detect individual rearrangements containing specific IGHV genes, naïve B-cell repertoires, defined as CD19^+^CD27^−^IgD^+^CD38^dim^CD24^+^ cells (Figure S1 in Supplementary Material), were analyzed at 40× depth per starting cell. We assumed that the naïve B-cell subpopulation had not yet encountered foreign antigen and hence would have not undergone clonal expansion. Therefore, a unique IGHV-D-J rearrangement would indicate the presence of a single cell in the sample of 40,000 cells analyzed per donor. Hence, we used the UMIG count per unique IGHV-D-J sequence—proxy of the number of mRNA molecules sequenced—as an indicator of the IGHV-specific detectability of the methodology.

**Figure 3:**
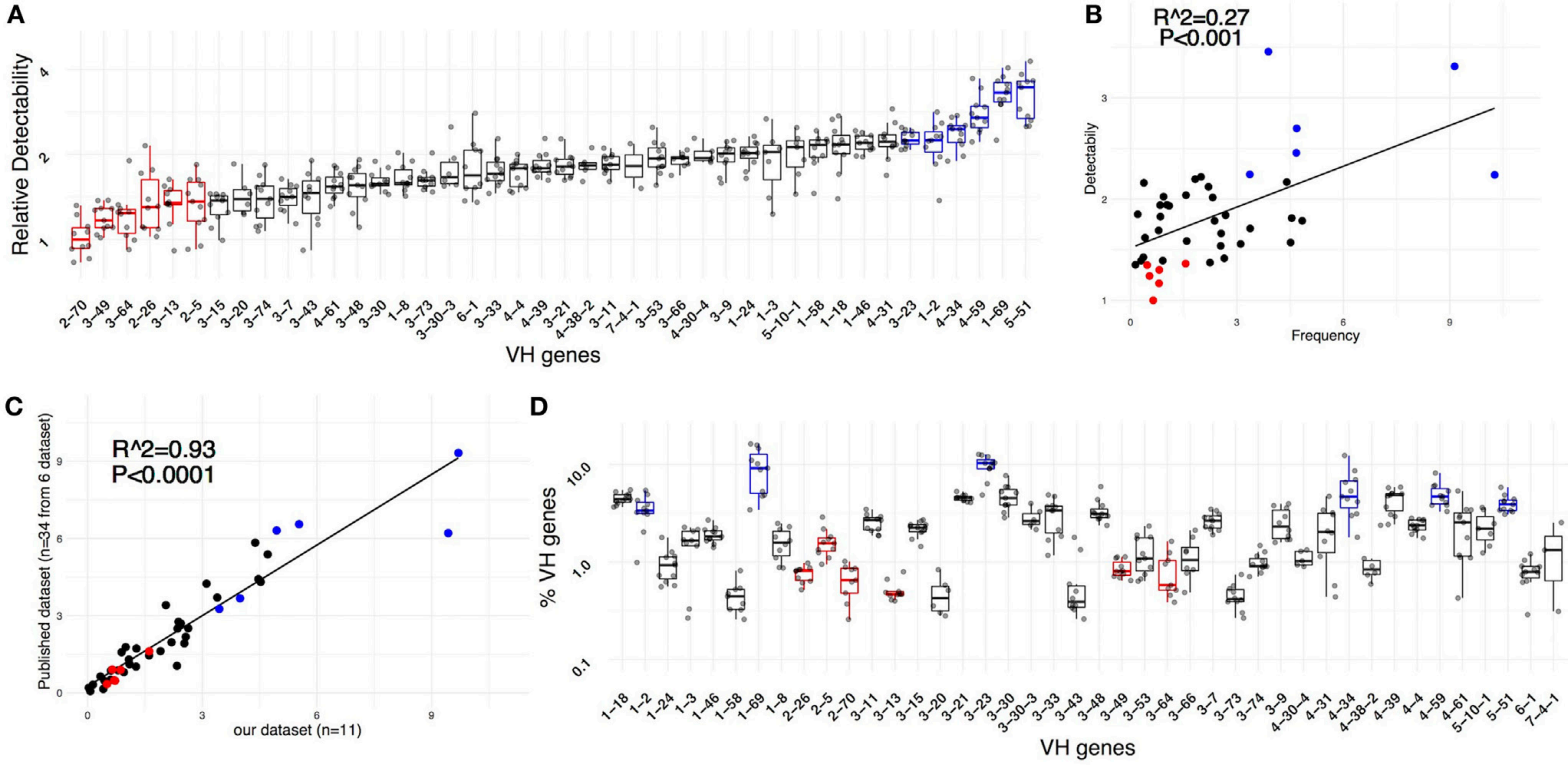
IGHV gene-specific detectability. **(A)** Relative detectability for all genes. For ease of comparison, the efficiencies were scaled to set the minimal median efficiency (corresponding to IGHV2-70) to 1. **(B)** The IGHV gene-specific detectability positively correlates with the IGHV gene frequency observed in unique sequences derived from naïve peripheral blood B cells (*R*^2^ = 0.27, *P* < 0.001). **(C)** The average IGHV gene frequency observed in our dataset (*n* = 11) correlates with the average frequency observed in 34 samples from 6 different published datasets (*R*^2^ = 0.93, *P* < 0.0001). **(D)** IGHV gene frequencies and intra-sample variability.

To quantify relative IGHV gene detectability, we compared the UMIG distribution of a specific gene to the distribution of all genes in the corresponding sample; this was done using a robust, non-parametric quantile-based method (see Methods) to estimate gene-specific detectability (Figure 3A). This indicated that, between the least and the most detectable IGHV gene, there was almost a fourfold difference, and detectability positively correlated with IGHV gene-use frequency observed (Figure 3B).

Since the least detectable genes were also the less frequent, we investigated if a lower detection efficiency biased the abundance by correlating our observed IGHV gene frequencies with those from PBMC-derived naïve B cells from six previously published datasets involving a total of 34 donors (Figure 3C; Figure S2 in Supplementary Material). Notably, the libraries used for sequencing in these studies were prepared by methods distinct from ours: multiplex PCR from Adaptive Biotechnologies (8), multiplex with primers on the FW1 (9–11), and RACE PCR (6, 12). In our dataset of 11 donors, we observed a relatively high intra-sample biological variability in the frequency of IGHV use (Figure 3D). Therefore, the data from different samples were averaged, thereby minimizing the effect of biologic- and method-specific variability. There was a very strong correlation of IGHV gene frequencies in our dataset with those in the six other sets (*R*^2^ = 0.93, *P* < 0.0001), suggesting that specific IGHV gene use defined in our library was not significantly biased. Thus, the IGHV frequencies for individual genes were not solely the consequence of low efficiency for specific alleles. In addition, since the calculated IGHV gene-specific detectability was proportional to the number of UMIs sequenced per unique rearrangement, the data suggest that in some instances—at least for naïve B cells—IGHV gene use and IGH mRNA expression might be connected. Therefore, the estimated IGHV gene use frequency observed might reflect, at least in part, a true, not yet described biological phenomenon.

### Use of Chronic Lymphocytic Leukemia (CLL) Cell Spike-In to Assess Sequencing Sensitivity

**Figure 4:**
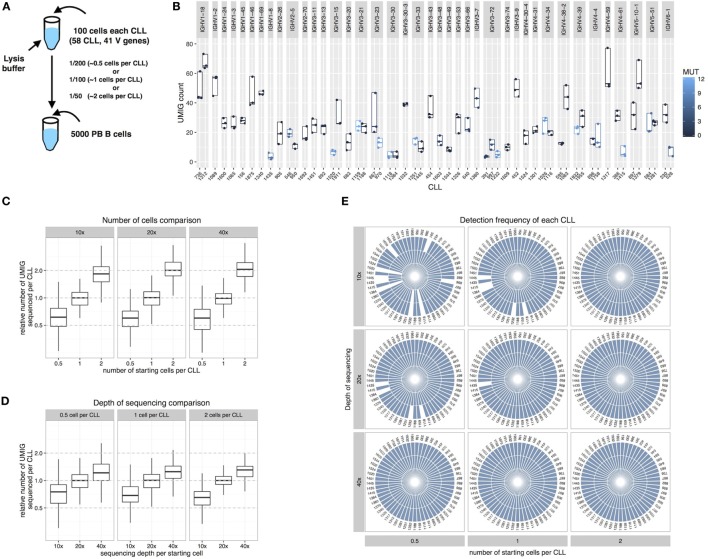
Chronic lymphocytic leukemia (CLL) spike-in experiments. **(A)** A cell lysate containing 100 cells sorted from 58 different CLL samples was added to a lysate of 5,000 PBMCs derived from a normal person. The diluted material represents the equivalent of 0.5, 1, or 2 cells per CLL. **(B)** UMI group (UMIG) count across three replicates for every CLL and IGHV gene. **(C)** Relative UMIG count relative to input material. **(D)** Relative UMIG count relative to depth of sequencing. **(E)** Each slice in the pie charts represents a different CLL, and splice size indicates the frequency of detection among the three replicates. The depth of sequencing is calculated as a multiple of the number of starting cells for the library preparation.

We investigated the extent that we could detect every individual IGHV-D-J rearrangement using the basic error correction and filtering approaches mentioned above. To do so, we spiked into a PBMC-derived polyclonal B cells population leukemic B cells from patients with CLL, a disease of clonal B lymphocytes presenting the morphology of resting B cells and with a known, discriminatory IGHV-D-J sequence. Specifically, 100 leukemic cells from 58 different CLL samples were sorted into a single tube containing cell lysis buffer. This collection represented 41 different IGHV genes, of which 37 were identical to the germline sequence and 21 exhibited somatic mutations with 1–10% differences from the corresponding germline sequence (Figure 4A; Table S2 in Supplementary Material). A fraction of the CLL lysate (1/200, 1/100, or 1/50 dilutions containing equivalent genetic material to 0.5, 1, or 2 CLL cells) was then mixed with a cell lysate created from 5,000 polyclonal B cells from a healthy donor. Using our Ig-seq method, we identified the CLL-specific rearrangements and assessed the presence of each of the 58 different CLL signatures in each condition/replicate. Since each B cell should contain multiple copies of its signature IGH mRNA, even at the higher dilution (0.5 equivalent cells per CLL) material from each CLL would be present and detectable.

#### Relative Detection Frequency per CLL

Each CLL spike-in (0.5, 1, or 2 cells) was performed in triplicate, and the resulting library sequenced independently at 10×, 20×, and 40× relative to the number of starting cells (i.e., for 5,000 starting cells, 40× equals ~200,000 raw sequences).

The UMIG count per CLL IGHV-D-J was highly variable across CLLs but consistent across replicates (Figure 4B); this possibly reflected different IGHV-D-J mRNA expression levels in individual samples and/or sequence-specific efficiency differences. On average, the UMIG count increased proportionally to the amount of starting genetic material (Figure 4C), and increasing the depth of sequencing only marginally affected the UMIG count (Figure 4D).

#### Impact of Sequencing Depth and B-Cell Type

An appropriate depth of sequencing is crucial to obtain complete coverage of the starting material and to allow appropriate error correction using the UMIs. Sophisticated error correction techniques (13) require a high depth of sequencing, and this results in greater cost to perform extensive studies with the current technology. Hence, the ideal parameter to use when choosing the depth of sequencing would be the number of starting molecules determined using digital PCR (5) or qPCR. However, a more practical approach is to consider the number of starting cells (6). Here, we focused on obtaining comprehensive coverage—with the basic error correction described above—by correlating the starting number of cells from discrete B-cell populations (i.e., naïve, memory, or plasma cells) quantified by FACS during cell sorting.

As expected, both the depth of sequencing and the amount of starting material per CLL (equivalent number of starting cells) influenced the ability to reproducibly detect each leukemic rearrangement within the healthy PBMC material (Figure 4E). Specifically, starting with two cells per CLL sample, a depth of 10× was sufficient for complete coverage; however for 1 and 0.5 cells, a depth of at least 40× was needed. Also, modulating the stringency of filtering led to a change in sensitivity (Figure S3 in Supplementary Material). For example, by choosing a read count of two sequences or higher, full CLL coverage from 1 cell at 20× depth was obtained; however, at a read count of 5 or higher, information for 0.5 cells at 40× depth was lost. Note that at 40× depth, in this experiment, we maintained full CLL coverage with read counts 7 or higher.

#### Impact of Genetic Material Dilution

**Figure 5:**
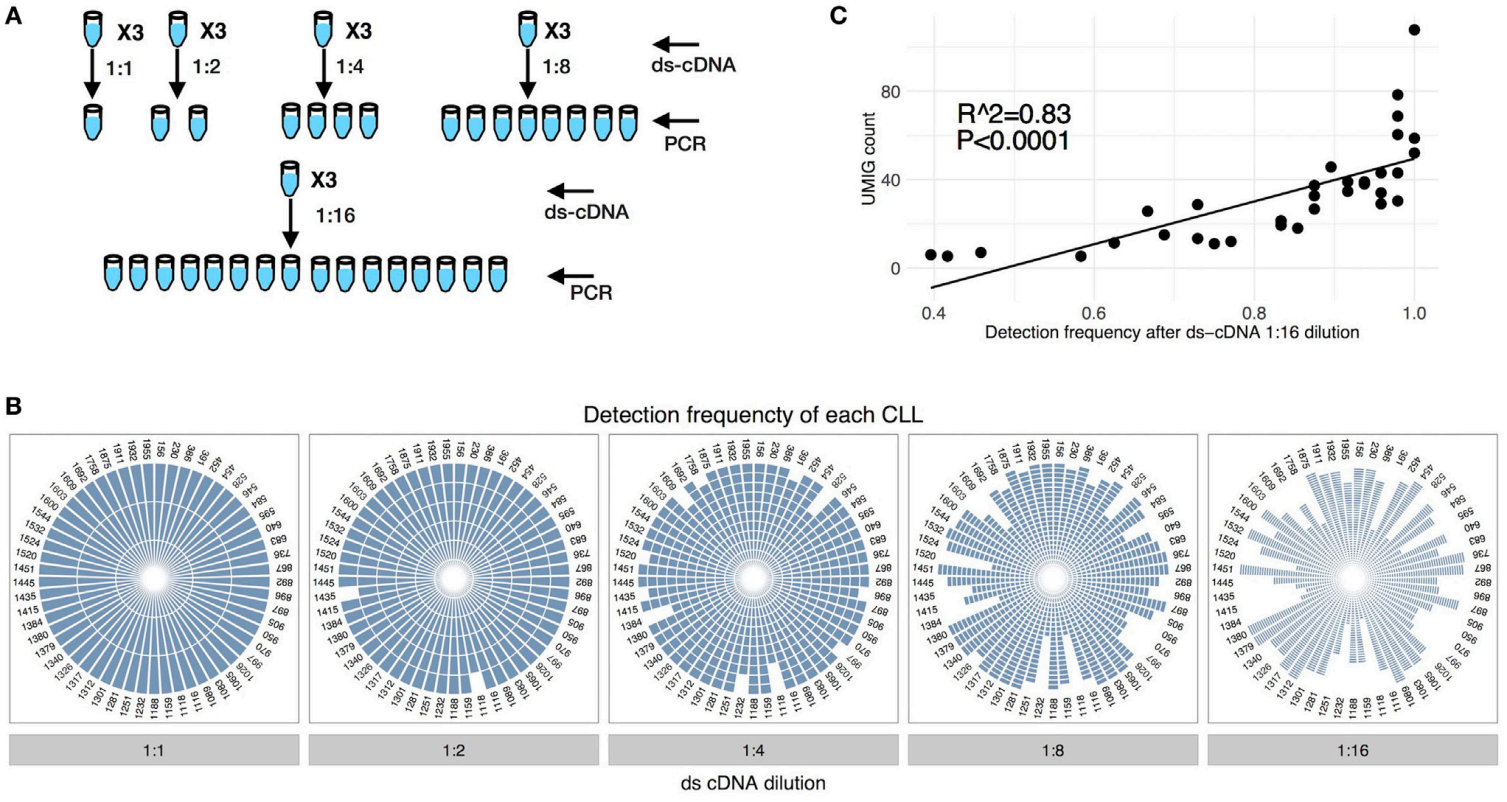
Identification of chronic lymphocytic leukemia (CLL) spike-in upon dilution. **(A)** CLL spike-in was performed as in Figure 4A. The ds-cDNA obtained from a CLL spike-in with 1 cell per CLL was diluted at different levels (1:1, 1:2, 1:4, 1:8, and 1:16) to simulate genetic material dilution before PCR amplification step. **(B)** Impact of the dilution of genetic material on the identification of specific CLL IGHV genes. Every fraction of the three different replicates was sequenced. **(C)** The UMIG count of CLL spike-in correlates with the detection frequency after 1:16 dilution.

We also used the spike-in data to assess the effect of genetic material dilution during library preparation on sequencing resolution. Lysate from PBMC spike-in with 1 cell per CLL was used to prepare ds-cDNA without genetic material dilution between steps, as described above. Then the ds-cDNA from each tube was divided into multiple aliquots at defined dilution factors (undiluted, 1:2, 1:4, 1:8, or 1:16). Each dilution was analyzed in triplicate, and each aliquot sequenced independently at depth 40× (Figure 5A). The results indicated that diluting the genetic material by only 50% compromised the ability to consistently detect each CLL IGHV-D-J (detected 56 out of 58 CLL); this deficiency became even more significant with further dilutions (Figure 5B). Thus, our use of mRNA, cDNA, and ds-cDNA purification using poly-T coupled magnetic beads was crucial. As expected, the UMIG count for each CLL correlated with the detection reproducibility upon genetic material dilution (Figure 5C).

## Discussion

We have devised a protocol for Ig-seq that reaches single cell resolution. Being able to sequence every cell in a sample is particularly important in studies involving non-expanded B-cell clones such as for analyses of B-cell development, naïve B lymphocytes, long-term immunological memory where the larger clonotypes occur in <0.5% of memory B cells in the peripheral blood (6), or minimal residual disease, where the IGV-D-J rearrangement of interest is by definition not frequent. The methodology is also applicable for less polyclonal repertoires. For example, in studies of intraclonal diversification in leukemia/lymphoma, this method allows the following of clonal evolution by detecting with high accuracy and sensitivity subclonal variants even when present at low frequency (manuscript in preparation).

Our methodology results in a highly efficient process that yields a comprehensive repertoire representation of the starting sample, even when this is as low as ~100 cells. Indeed, we show for the first time the importance of circumventing genetic material dilution in the pre-amplification phases. Capturing mRNA from the entire cell lysate on poly(dT) magnetic beads and then carrying out cDNA synthesis on the same solid phase leads to yields that are several fold higher than conventional approaches (data not shown). This is because in most sequencing protocols, only a fraction of the original genetic material contributes to the resulting library due to dilution of the extracted mRNA and/or the cDNA prior to PCR amplification.

Overall, our data agree with the mRNA quantification estimates for plasma cells, memory, and naive B cells reported by Turchaninova et al. (500:5:2). Thus, theoretically, when dealing with B-cell populations with higher IGH mRNA content than naïve B cells (e.g., memory B cells and plasma cells), the starting genetic material might require dilution based on IGH mRNA content relative to that in naïve cells. However, for memory B cells, diluting the mRNA did not have major impact on the resulting sequences, leading only to a shift in UMIG and raw reads count per unique sequence (data not shown). This was not the case for plasma cells that contain more than 100 time mRNA, indicating the requirement for tight control of the amount of B cells from which mRNA is collected. With 40× depth per starting cell, ~500,000 B cells can be sequenced in a single MiSeq run, although the system is calibrated to work with a range from a few hundred up to tens of thousands of cells per reaction tube (data not shown).

Moreover, by introducing a universal forward priming site during the ds-cDNA synthesis [as done by Vollmer et al. (4)], we reduced a potential bias that can occur when employing multiplex primers that undergo exponential PCR amplification. In this regard, we measured the differential detectability of IGHV-D-J rearrangements containing different IGHV genes. This indicated a relative IGHV gene-specific detectability of approximately fourfold. Notably, at least part of these differences appeared to reflect true *in vivo* biology and not solely the consequence of a technical artifact. Although methodological biases can come into play—such as those occurring as a consequence of the multiplex approach for the ds-cDNA synthesis—differential IGH mRNA content should be considered. This latter possibility requires further investigation to assess and understand the extent of these phenomena.

We chose to filter out sequences with read counts less than three. This threshold provided sequences where basic error correction was performed, without a major loss in sensitivity since monoclonal B cell spike-in experiments indicated that we could increase the threshold up to sevenfold without losing information (Figure S1 in Supplementary Material). The latter might not be true for B cells with very low IGH mRNA content, in which case a lower threshold might be preferable, although this might artificially increase diversity. Overall, using sequences with read counts three or higher, at 40× sequencing depth per starting cell provided error corrected sequences with good coverage at a reasonable cost. Increasing coverage and using more sophisticated error correction methods will give a more reliable dataset, compatible with our protocol.

Every sequencing platform and library preparation protocol results in a certain level of error. For this reason, allowing errors in the UMI region is becoming common practice (2, 5, 14). However, we observed that more important than possible errors in the UMI region is the level of UMI diversity. Even with a limited starting population of only 5,000 naïve B cells per reaction and a theoretical diversity between 4^13 and 4^16 (depending on the specific primer in the multiplex), the apparent UMI diversity was insufficient. Within each UMI cluster, we created sub-clusters based on sequence identity. This led to a striking 30% increase in the number of sequences passing the filter of ≥3 raw reads per unique sequence. The approach taken by Khan et al. (5), where the UMI design does not follow a simple NNN… pattern, might mitigate the problem of the decreased real UMI diversity reducing the complexity of the UMI nucleotide sequences.

In conclusion, we have developed a protocol for Ig-seq where virtually every IGHV-D-J rearrangement in the starting B-cell population(s) can be detected. To achieve this result, we used a methodology with an overall efficiency sufficient to retain the “full” repertoire diversity of the sample analyzed. The key aspects of the method consist in starting from a defined number of cells for which one wants to know the repertoire, avoiding primer-specific PCR amplification and dilution of the starting genetic material for low IGH mRNA content cells, and achieving a minimum of 40× depth per starting number of cell.

## Methods

### Samples

The study was approved by the Institutional Review Board of Northwell Health. Written, informed consent was obtained before blood collection from CLL patients in accordance with the Declaration of Helsinki. PBMCs from the CLL patients and from anonymous healthy blood donors were separated by density gradient centrifugation (Ficoll, GE Healthcare), frozen (10% DMSO, 45% FBS, and 45% RPMI), and stored in liquid nitrogen until used.

### Cell Sorting

PBMCs from normal blood donors were incubated with the following anti-human Abs: V500 anti-CD19 (BD Biosciences), PerCPcy5.5 anti-CD38 (BioLegend), PE-cy7 anti- CD24 (BioLegend), FITC anti-IgD (ThermoFisher), and allophycocyanin anti-CD27 (BD Bioscien). CLL patient PBMCs were exposed to the following anti-human Abs: V500 anti-CD19 (BD Biosciences) and PE-cy7 anti-CD5 (Invitrogen). Non-B cells were excluded with efluor-450 anti-CD3 and anti-CD16, and dead cells were excluded by Sytox Blue staining (ThermoFisher). B cells were sorted directly into 200 µl PCR tubes containing 100 µl Dynabeads Oligo(dT) (ThermoFisher) lysis buffer and stored at −80°C.

### Library Preparation and Sequencing

mRNA isolation from B-cell lysates was performed using Dynabeads mRNA DIRECT Micro Kit (ThermoFisher). The protocol used was that suggested by the manufacturer, except that mRNA isolation was performed in 200 µl 96-well PCR plates to enable parallel processing with the support of a 96-well magnetic stand. mRNA was used in its entirety for reverse transcription in 10 µl (50°C 1 h, 72°C 10 min) using SuperScript III Enzyme (ThermoFisher) in solid phase with Dynabeads Oligo(dT) as primer. After RNase H treatment, second-strand synthesis was performed in solid phase in 10 µl using Q5 Polymerase (NEB) and a mix of 13 primers covering all IGHV leader sequence segments reported in the IMGT database with a maximum of one mismatch, containing 13 to 16 random nt and partial Illumina adaptor sequences (37°C 20 min, 98°C 30 s, 62°C 2 min, and 72°C 10 min). Double-stranded cDNA was washed three times in 10 mM tris–HCl to remove the remaining primers, and the entire sample was used as template for PCR amplification in 10 µl using Q5 Polymerase with universal FW primer and mix of reverse isotype specific primer (98°C 30 s; 10 cycles of 98°C 10 s, 58°C 15 s, and 72°C 1 min; 72°C 10 min). Two microliters of the PCR product were used for a semi-nested PCR with inner RV primers for the constant region, which also introduce partial Illumina adaptors. This reaction was carried in 20 µl (98°C 30 s; 15 cycles of 98°C 10 s, 58°C 15 s, and 72°C 1 min; 72°C 10 min). The PCR product was purified with Ampure XP beads at a ratio of 1:1, and 1–10 ng used to add Illumina Index with Nextera XT kit (Illumina). The MiSeq Illumina (v3 2 × 300 kit, Illumina MS-102-3003) was used to sequence the library. The library was loaded at 12 pm with 10% PhiX. The list of the primers is in Table S1 in Supplementary Material. Raw data are deposited at SRA (BioProject ID PRJNA381394—http://www.ncbi.nlm.nih.gov/bioproject/381394).

### Bioinformatic Analysis

Processing of raw reads was performed using a custom workflow built with pRESTO (REpertoire Sequencing TOolkit) (7). IGHV sequences obtained were then submitted to IMGT/HighV-QUEST (15) and analyzed using ChangeO (16), and custom R scripts.

### Relative Detectability Estimation

A metric to quantify the relative abundance of gene-specific UMIG counts and to compare this to the total abundance of all IGHV genes within a sample was developed. In order to focus on gene-specific patterns, relative measure was used. Starting with two UMIG count distributions, the relative abundance metric summarizes the position of one distribution relative to the other. Each distribution was encoded as a vector of fine grained quantiles and performed a linear regression between the paired sets of quantiles of the two distributions. The slope of this line represents the relative shift of one distribution against the other and was thus termed the relative efficiency. In this paper, were used 100 quantiles, from 0 to 99, evenly spaced at 1% intervals for the detectability estimation. For this reason, were discarded any distributions with fewer than 100 points from the analysis. Figure S4 in Supplementary Material shows intermediate steps of this analysis for a highly detectable gene (IGHV5-51) and a low detectable gene (IGHV2-70), across all samples.

## Ethics Statement

This study was carried out in accordance with the recommendations of the Belmont Report, and the Office of Human Research Protection Program Institutional Review Board at Northwell Health System. All CLL samples were from individuals who provided written informed consent for the collection and use of samples for research purposes according to the Declaration of Helsinki. The Protocol was approved by the Northwell Health Institutional Review Board.

## Author Contributions

DB and SV performed the experiments; DB, SV, and IK analyzed the data; NC provided project funding; DB, SV, AM, GF, and NC interpreted the results and wrote the manuscript; DB designed the experiments and directed the project.

## Conflict of Interest Statement

The authors declare that the research was conducted in the absence of any commercial or financial relationships that could be construed as a potential conflict of interest.
