# Supplementary material for: Novel Method for High-Throughput Full-Length IGHV-D-J Sequencing of the Immune Repertoire from Bulk B-Cells with Single-Cell Resolution
Source: Front Immunol. 2017 Sep 14;8:1157. doi: 10.3389/fimmu.2017.01157 (PMC5603803; doi:10.3389/fimmu.2017.01157)
Supplement: Supplementary file 1 [file Table_1.DOCX]

Table S1

| Primers name | Primers sequence |
| --- | --- |
| ds-cDNA synthesis | |
| L3_VH1* | GCGTCAGATGTGTATAAGAGACAGNNNNNNNNNNNNNNNCAACTACAGGTGCCCACTCC |
| L3_VH1-46 | GCGTCAGATGTGTATAAGAGACAGNNNNNNNNNNNNNNTAGCTCCAGGTGCTCACTCC |
| L3_VH1-69 | GCGTCAGATGTGTATAAGAGACAGNNNNNNNNNNNNNNNNCAGCYACAGGTGTCCASTCC |
| L3_VH1-2 | GCGTCAGATGTGTATAAGAGACAGNNNNNNNNNNNNNNNCMACAGGWGCCCACTCC |
| L3_VH1-45 | GCGTCAGATGTGTATAAGAGACAGNNNNNNNNNNNNNNAGCCACAGATGCCTACTCC |
| L3_VH1-24 | GCGTCAGATGTGTATAAGAGACAGNNNNNNNNNNNNNCTACAGGCACCCACGCC |
| L3_VH2 | GCGTCAGATGTGTATAAGAGACAGNNNNNNNNNNNNNNCCKTCCTGGGTCTTRTCC |
| L3_VH2-70*09 | GCGTCAGATGTGTATAAGAGACAGNNNNNNNNNNNNNNNCCTTCATGGGTCTTGTCT |
| L3_VH3* | GCGTCAGATGTGTATAAGAGACAGNNNNNNNNNNNNNNNTTWAAAGGTGTCCAGTGTGARG |
| L3_VH3-30/33/11 | GCGTCAGATGTGTATAAGAGACAGNNNNNNNNNNNNNWTAARAGGTGTCCAGTGTCAGG |
| L3_VH4 | GCGTCAGATGTGTATAAGAGACAGNNNNNNNNNNNNNNNCCAGATGGGTCCTGYCC |
| L3_VH5-51 | GCGTCAGATGTGTATAAGAGACAGNNNNNNNNNNNNNNNTTCTCCAAGGAGTCTGTKCC |
| L3_VH6-1 | GCGTCAGATGTGTATAAGAGACAGNNNNNNNNNNNNNNNCCATGGGGTGTCCTGTCA |
| PCR1 and PCR2 FW primer | |
| IL-F1 | TCGTCGGCAGCGTCAGATGTGTATAAGAGACAG |
| PCR1 RV primers | |
| CHM^1^ | CAGGAGACGAGGGGGAAAAGG |
| CHA^1^ | GGAAGAAGCCCTGGACCAGGC |
| HCG | TTCGGGGAAGTAGTCCTTG |
| PCR2 RV primers | |
| HCA-n2m | GTCTCGTGGGCTCGGAGATGTGTATAAGAGACAGGGGAAGACCTTGGGGCTGGT |
| HCG-n2m | GTCTCGTGGGCTCGGAGATGTGTATAAGAGACAGGGAAGACCGATGGGCCCTT |
| HCM-n2m | GTCTCGTGGGCTCGGAGATGTGTATAAGAGACAGAAAGGGTTGGGGCGGATGC |

^1^ Y.-C.B. Wu, D. Kipling, H.S. Leong, V. Martin, A.A. Ademokun, and D.K. Dunn-Walters, Blood **116**, 1070 (2010).
