# Supplementary material for: Novel Method for High-Throughput Full-Length IGHV-D-J Sequencing of the Immune Repertoire from Bulk B-Cells with Single-Cell Resolution
Source: Front Immunol. 2017 Sep 14;8:1157. doi: 10.3389/fimmu.2017.01157 (PMC5603803; doi:10.3389/fimmu.2017.01157)
Supplement: Supplementary file 2 [file Table_2.DOCX]

Table S2

| CLL_ID | IGHV gene | IGHV mutation | CDR3 |
| --- | --- | --- | --- |
| 1089 | 1-02 | 0% | TGTGCGAGAGGGCAGTGGCTGCCCACTTTATCCTTTGACTACTGG |
| 1065 | 1-03 | 0% | TGTGCGAGAGAGGGGTTTTATGATTACGTTTGGGGGAGTTATCGTCTCCGCGGTGGGTACTACTTTGACTACTGG |
| 1435 | 1-08 | 7% | TGTGCGAGAGCCCCGGGACGGCGCTGTAGTGGTGGAAGTTGCTACTCAAGTTTCCGGTGGTTCGACCCCTGG |
| 1312 | 1-18 | 0% | TGTGCGAGAGAGCAGTGGCTGCCAATCGACTACTTTGACTACTGG |
| 1600 | 1-24 | 0% | TGTGCAACACCGCGGCTACGATTCTCTTACTACTACTACTACGGTATGGACGTCTGG |
| 736 | 1-28 | 0% | TGTGCGAGAGACCCGGATATTGTAGTAGTACCAGCTGCTCCATATTACTACTACTACTACGGTATGGACGTCTGG |
| 156 | 1-45 | 0% | TGTGCATTGGCCGAAAGTAGTACCAGCTGCATAAACAGTGTCTGTATCACCCCAGAAACAGGTAGCTGGTTCGACCCCTGG |
| 1875 | 1-46 | 0% | TGTGCGAGAGATAGTCGGTATTGTAGTAGTACCAGCTGCTATGTTGGGCGGGACTGGTTCGACCCCTGG |
| 1340 | 1-69 | 0% | TGTGCGAGCCGCGATATTACGATTTTTGGAGTGGTTATTATAAGGGGCTACTACTACTACGGTATGGACGTCTGG |
| 546 | 2-05 | 7% | TGTACACACAGACCAGATTGTAGTGTTGGTCACTGCTACGATGACTACTGG |
| 950 | 2-05 | 0% | TGTGCACACAGCGGAAGCAGTGGCTGGTATGAGGTTCCGGAGCGGCACTACTACTACTACGGTATGGACGTCTGG |
| 905 | 2-26 | 0% | TGTGCACGGATTCACGTCACCTGGGCCGATTTTTGGAGTGGACCCAATTACTACTACTACTACGGTATGGACGTCTGG |
| 1692 | 2-70D | 0% | TGTGCACGGTCATCTAACTGGGGATCCACCGGCTTTGACTACTGG |
| 1380 | 3-07 | 3% | TGTGCGAGACCTTCCGCCCCCGGTGACTACGATGCTCTTGATATCTGG |
| 452 | 3-09 | 0% | TGTGCAAAAGATCATTACGATTTTTGGAGTGGTTATCCAAACCTCTCTCCCTTCGATCTCTGG |
| 1451 | 3-11 | 0% | TGTGCGAGAAAACACATATCCAATTACTATGATAGTAGTGGTTATTACTACATGGGCTACTACTACGGTATGGACGTCTGG |
| 892 | 3-13 | 1% | TGTGCAAGAGCAGTCTCGACTACGGTGGGGGACCCTCCGGGCTACTGG |
| 1520 | 3-15 | 10% | TGTACCACAGATTTGGCAACACCGAGCCCCCCGGATGGTCGTGATTACTTTGACCACTGG |
| 1911 | 3-15 | 0% | TGTACCACAGATCTAGGGTACTATGATAGTAGTGGTTATTACTACGTGAATTGGGCTGAATACTTCCAGCACTGG |
| 683 | 3-20 | 0% | TGTGCGAGAGAGAGGAATGCGTATTACGATTTTTGGAGTGGTTATTACACCCAGTACTACTTTGACTACTGG |
| 1159 | 3-21 | 12% | TGTGCGAGAGAAATGAGTAACGAAGGGTTTGACTACTGG |
| 1188 | 3-21 | 0% | TGTGCGAGAGATTTGTTCTACTATGATAGTAGTGGTTATTATTCGGGTTTTTTTGACTACTGG |
| 867 | 3-23 | 2% | TGTGCGAAAGGAGTCTATTGTAGTAGTACCAGTTGCTATGAGGGTCGTGGGTACTACTACGGTATGGGCGTCTGG |
| 970 | 3-23 | 11% | TGTGCGAAACTCCCAAGTGGGAGGATAGTGGGGCCTCTCTACTACTTTGACTCCTGG |
| 1118 | 3-30 | 10% | TGTGCGAAAGGGCATAGTGGTCAGATTGGAAGTCCCCACGACTATTACTTTGACTATTGG |
| 1384 | 3-30 | 0% | TGTGCGAAAGATCCCTCCTCCCCTACAGTAACCTCTGATTACTACTACTACTACGGTATGGACGTCTGG |
| 1532 | 3-30-3 | 0% | TGTGCGAGAGGAGCGAGCGGTGACTACGTATCGTCCTTTGACTACTGG |
| 1251 | 3-33 | 11% | TGTGTGAGAGAGCGTGCACCCTTTGATGCTTTTGATGTCTGG |
| 1445 | 3-33 | 0% | TGTGCGAGATTCTCCCGGCCGTATTACGATTTTTGGAGTGGTTATTATGTTACGGACTACTACTACATGGACGTCTGG |
| 454 | 3-43 | 0% | TGTGCAAAAGATATCAGTGGCTGGCCCGAATATTACTACTACTACTACGGTATGGACGTCTGG |
| 1603 | 3-48 | 0% | TGTGCGAGAGATTCCCCCCTGGTAGTACCAGCTGCTATCTTTTACTACTACTACGGTATGGACGTCTGG |
| 1544 | 3-49 | 0% | TGTACTAGAGATGCCGTAACTATCTGGGAGCATATTGTGGTGGTGACTGCACCACCAGTTTATTACTACTACTACTACGGTATGGACGTCTGG |
| 1326 | 3-53 | 0% | TGTGCGAGAGATCGGGTGGATATAGTGGCTACGACTACCTATTACTACTACTACTACGGTATGGACGTCTGG |
| 640 | 3-66 | 2% | TGTGCGAGTAGTAAGGACCCAGGCACATTTGACTACTGG |
| 391 | 3-72 | 1% | TGTGCTAGGTTAGGATATTGTAGTAGTACCACTTGCCGACAAGCTTTTGATATCTGG |
| 997 | 3-72 | 4% | TGTGTCAGAGCTAGAGTTTGTGGTGGTGGTAGGTGCACTCCCAACATGGACGTCTGG |
| 1232 | 3-72 | 10% | TGTGCTAGAGGAGAGACTGGGGTGACTGACCCAATAGAAATGATCTTCGACGCCTGG |
| 1609 | 3-74 | 0% | TGTGCAAGAGACCTACTCCCTAATTACGATTTTTGGAGTGGTTATTCTCACGCATACTACTACTACTACGGTATGGACGTCTGG |
| 896 | 4-04 | 0% | TGTGCGAGATTTCTAACTGGAACGACTATCTACTACTACTACGGTATGGACGTCTGG |
| 1758 | 4-04 | 8% | TGTGCGAGTAATCCGCGTCTTGGGGGGGATGTTGTTGACAGCTGG |
| 1524 | 4-30-4 | 0% | TGTGCCAGAGTCCGTCGAGATGATAGTAGTGGTTATTACTACTACTACGGTATGGACGTCTGG |
| 1301 | 4-31 | 0% | TGTGCGAGAGCGCCCATAGGGTCTACGATTTTTGGAGTGGTTATTATACGTTTTGCTTTTGATATCTGG |
| 1026 | 4-34 | 10% | TGTGCGAGACGAGCAGAAAGGTGGGAGACGCTTCTTCGTGATGATTTTGATGTTTGG |
| 1116 | 4-34 | 0% | TGTGCGAGAGTCCAAGAACTTTACGATTTTTGGAGTGGTTATCGCGTACGGTACTACTACTACATGGACGTCTGG |
| 595 | 4-38-2 | 0% | TGTGCGAGACTACGAGATTTTTGGAGTGGTTATTATACCGGTTCAGATGCTTTTGATATCTGG |
| 1083 | 4-38-2 | 0% | TGTGCGAGAGATTCGGGGGGTTACGTTTGGGGGAGTTATGAGGCCTGGTTCGACCCCTGG |
| 1932 | 4-39 | 11% | TGTGCGAGACATGACCGTAAGACTTATACTGCGACGATGGGATGGTATGACTACTGG |
| 1955 | 4-39 | 0% | TGTGCGAGACGCACTACTATGGTTCGGGGAGTTCTTGAAATCGCGAGGTACTACTTTGACTACTGG |
| 1317 | 4-59 | 0% | TGTGCGAGGAACCCATATTGTGGTGGTGACTGCTATTCGGATGCTTTTGATATCTGG |
| 386 | 4-61 | 0% | TGTGCGAGAGTCGGGTCCACGACTACGGTGGTAACTCCGGGTGGGAACTACTACTACTACGGTATGGACGTCTGG |
| 1415 | 4-61 | 10% | TGTGCGAGTACATTTTGTGGTGGTGAATGCTACTATGACCACTGG |
| 897 | 5-10-1 | 0% | TGTGCGAGAGTAGGATGGTCATACTATGATAGTAGTGGTTATTACTACGCCCCGGGTGGCTGGTTCGACCCCTGG |
| 1379 | 5-10-1 | 0% | TGTGCGAGACAGGTAGCAGTGGCTGGTAAATGGTGGGGTCCGTACTACTACTACGGTATGGACGTCTGG |
| 584 | 5-51 | 6% | TGTGCGAGACTGGGTGACGGTTATTATTATCATGGTTTTGATATCTGG |
| 1281 | 5-51 | 0% | TGTGCGAGACATCGAGGGTGGGGCTTAGTGGTGGTAGCTGCTACTCCATGGTACTACTACTACGGTATGGACGTCTGG |
| 230 | 6-01 | 0% | TGTGCAAGAGAGTGGGAGCAGCAGCTGTCCAACTGG |
| 528 | 6-01 | 5% | TGTGTCCGCGGCTTCTTCTTCCTCGGGATGGACGTCTGG |
