# Supplementary figures and images for: Novel Method for High-Throughput Full-Length IGHV-D-J Sequencing of the Immune Repertoire from Bulk B-Cells with Single-Cell Resolution
Source: Front Immunol. 2017 Sep 14;8:1157. doi: 10.3389/fimmu.2017.01157 (PMC5603803; doi:10.3389/fimmu.2017.01157)

## Supplemental Figure and tables

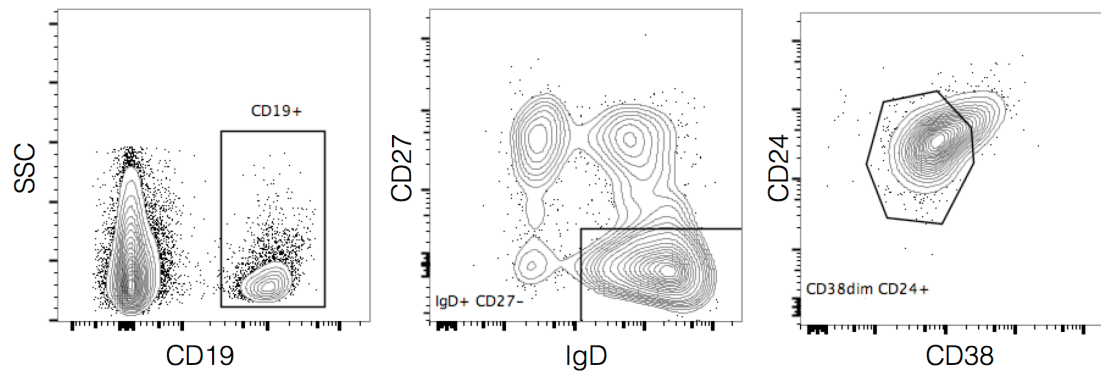

**Figure S1.** Gating strategy for Naïve B cell sorting

Supplement: Supplementary file 3 [file Image_1.PDF]

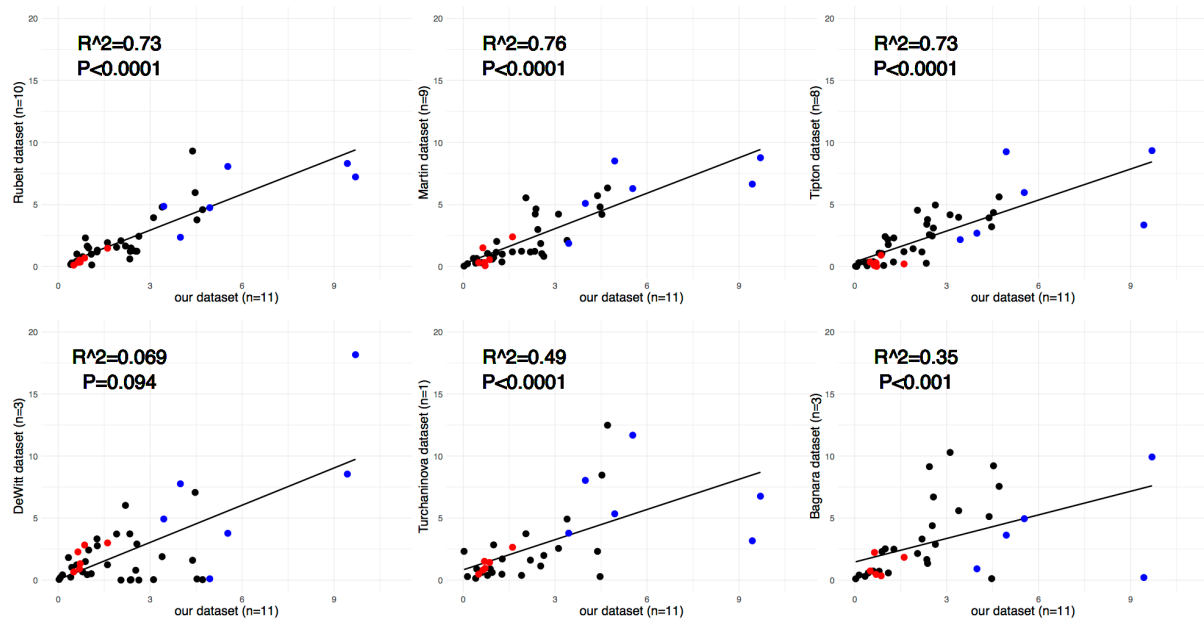

**Figure S2.** Observed IGHV gene frequency in naïve B cells (n=11) compared other 6 published datasets.

Supplement: Supplementary file 4 [file Image_2.PDF]

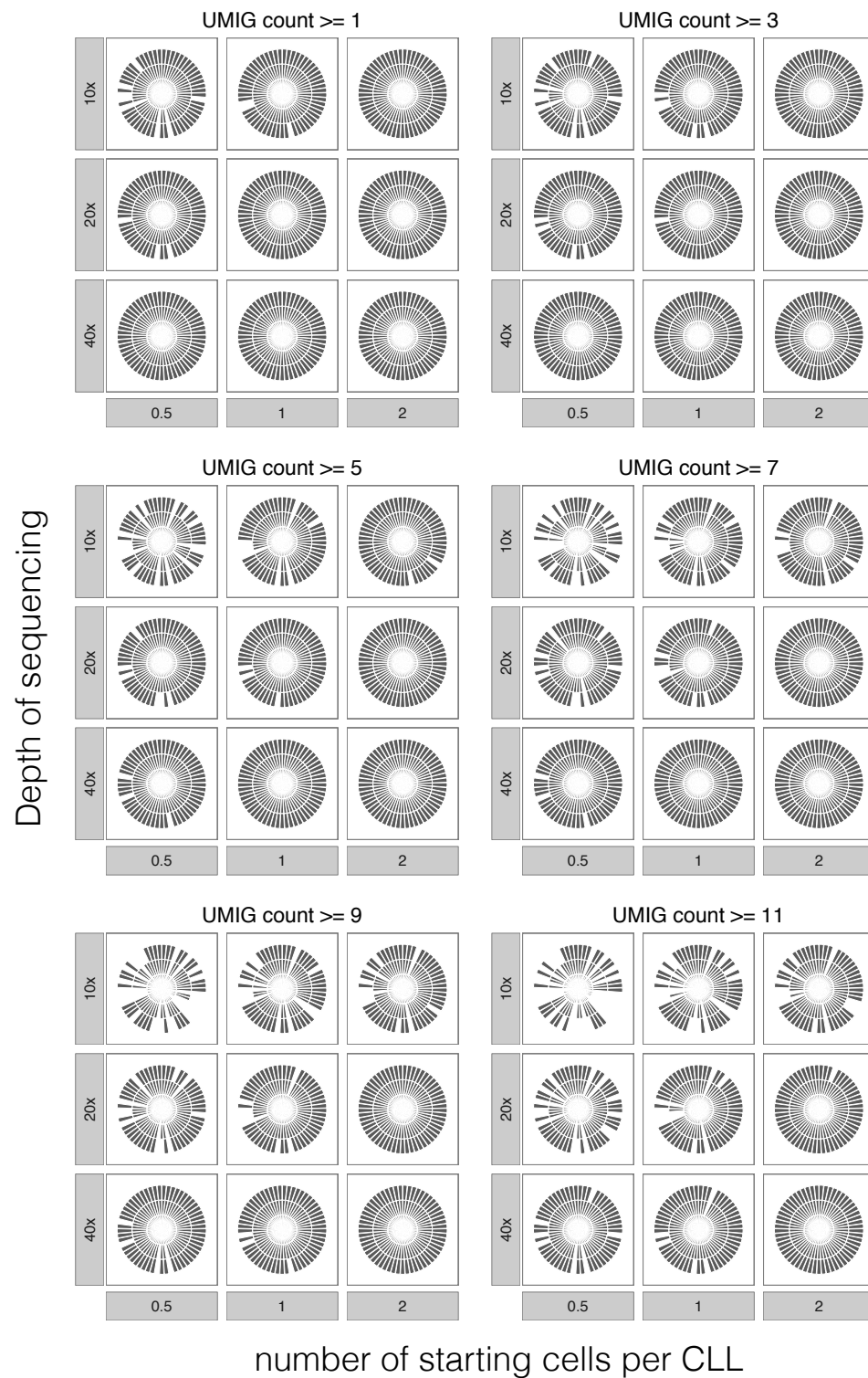

**Figure S3.** CLL spike-in detectability at different UMIG count filters.

Supplement: Supplementary file 5 [file Image_3.PDF]
