# Supplementary material for: Novel Method for High-Throughput Full-Length IGHV-D-J Sequencing of the Immune Repertoire from Bulk B-Cells with Single-Cell Resolution
Source: Front Immunol. 2017 Sep 14;8:1157. doi: 10.3389/fimmu.2017.01157 (PMC5603803; doi:10.3389/fimmu.2017.01157)
Supplement: Supplementary file 6 [file Image_4.PDF]

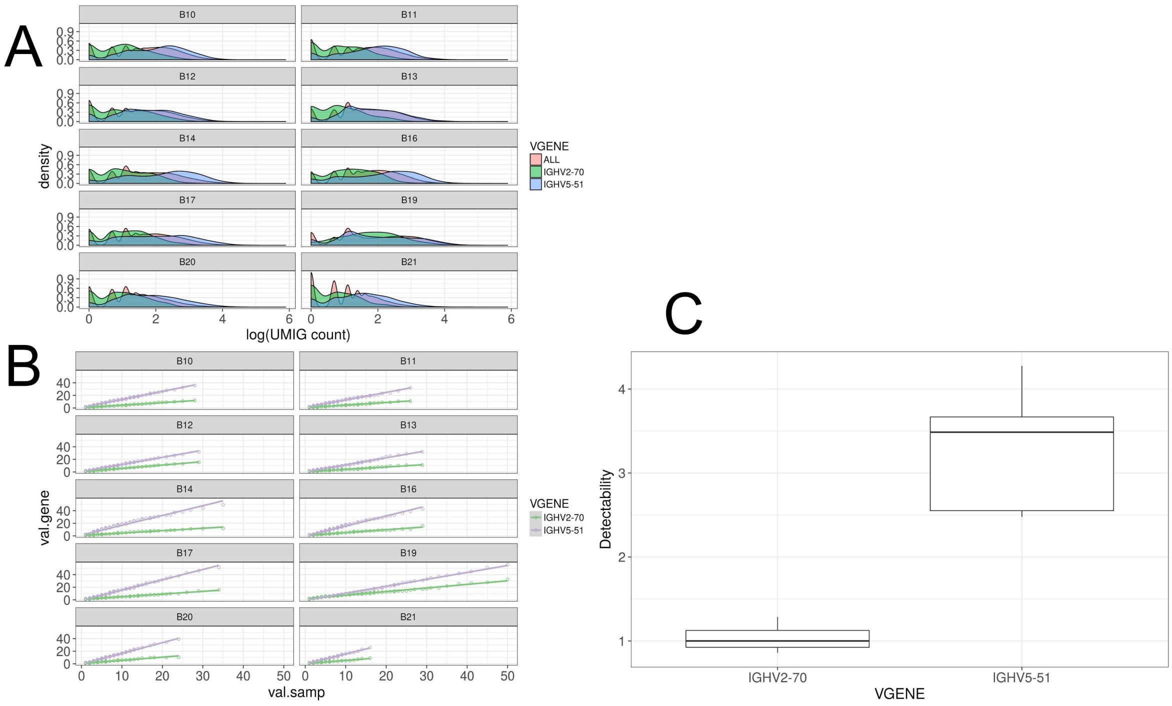

Figure S4. Demonstration of the relative detectability estimation procedure. Two genes were chosen with different degrees of average detection, IGHV5-51 and IGHV2-70. (A) The UMIG count distributions of each of these two genes are compared to those of the overall UMIG count, for all genes in the corresponding sample. Each subplot represents a distinct sample. Note that IGHV2-70 was not sufficiently detected in sample B18 to be included in the analysis. Visually, the blue density, which corresponds to IGHV5-51, is on average shifted further right than the green density, which corresponds to IGHV2-70. (B) The quantile-quantile plots of each gene against the overall gene counts. Each subplot compares 100 fine grained quantiles. Many of the nearby quantiles are identical and thus result in overlapping points in the plot. The greater detection of IGHV5-51 is now evident in the higher slopes of the purple regression lines than those of the green ones. (C) The slope of each regression line from (B) is computed for each sample and summarized in a boxplot. The median value of each boxplot defines the relative detectability of that gene.
